# The first metatarsal pronation leads to increased distal metatarsal articular angle: a self-controlled study

**DOI:** 10.1186/s12891-025-09306-3

**Published:** 2026-03-14

**Authors:** Jie Luo, Xiang Geng, Xu Wang, Xin Ma

**Affiliations:** 1 Department of Orthopaedics, Huashan Hospital, Fudan University, Shanghai, 200040 P. R. China; 2 Department of Orthopaedics, Shanghai Sixth People’s Hospital Affiliated to Shanghai Jiao Tong University School of Medicine, Shanghai, 200233 P. R. China

**Keywords:** Hallux valgus, Distal metatarsal articular angle, First metatarsal rotation, Sesamoid bone

## Abstract

**Objective:**

Our study aimed to analyze the relationship between distal metatarsal articular angle (DMAA) and first metatarsal (M1) pronation by measuring the DMAA and first metatarsal rotation angle (MRA) of patients with unilateral hallux valgus (HV) and discussed its significance in the surgery for HV treatment.

**Methods:**

We performed a retrospective self-controlled study including patients with unilateral HV from January 2015 to December 2018 in our hospital. The affected and contralateral normal feet were divided into HV and normal groups. The hallux valgus angle (HVA), DMAA, the first and second intermetatarsal angle (IMA), first metatarsal rotation angle (MRA), Hardy score in weight-bearing anteroposterior radiographs of the foot, and Yildirim score in tangential radiographs of the sesamoid were measured in the two groups. Statistical analysis was conducted to investigate the difference in all radiographic parameters between the two groups and the correlation between DMAA, Hardy score, and Yildirim score.

**Result:**

A total of 20 unilateral HV patients, including 1 man and 19 women (average age 53.35 ± 13.90, range 25–73), were enrolled in this study. The average HVA, DMAA, IMA, Hardy score, and Yildirim score in the HV group (*n* = 20) were 40.04 ± 8.96°, 29.40 ± 9.73°, 13.34 ± 2.73°, 5.5 ± 0.97, and 2.35 ± 0.65, respectively. The average HVA, DMAA, IMA, Hardy score, and Yildirim score in the normal group (*n* = 20) were 16.06 ± 2.70°, 16.13 ± 7.77°, 10.02 ± 2.14°, 3.4 ± 1.02, and 0.85 ± 0.73, respectively. There was a significant difference in DMAA between the two groups (*P* < 0.001). A significant positive correlation was observed between MRA and DMAA (*r* = 0.617, *P* = 0.004). However, no significant correlations were found between MRA and the Hardy score (*r* = 0.028, *P* = 0.908) or Yildirim score (*r* = 0.285, *P* = 0.223).

**Conclusion:**

M1 rotation is significantly correlated with DMAA in HV deformity, underscoring the importance of assessing rotational alignment in preoperative planning. Intraoperative correction of rotational deformity may be essential in cases of an inadequately corrected DMAA.

## Introduction

 In 1960, Piggot [14] introduced the concept of distal metatarsal articular angle (DMAA) firstly, suggesting that the increase in DMAA was due to increased hallux valgus angle (HVA) or deformation of the metatarsal head, and the fatigue of soft tissue caused by repetitive stress and forefoot rotation, including periarticular ligaments and joint capsules, also affected DMAA [2, 15]. Subsequent studies have concluded that the correction of DMAA is one of the important purposes of hallux valgus (HV) surgery. Even some researchers believe that DMAA must be considered for preoperative planning, and poor recovery of DMAA during the procedure is a crucial reason for the recurrence of HV and stiffness of the first metatarsophalangeal joint after surgery [16, 19].

In a cadaver study, Robinson et al. [15] found that DMAA changed 10° at 30° metatarsal pronation. In addition, degeneration of the articular surface also leads to a rise in DMAA, progressing at a rate of 1–3° per decade, with an average increase of 4.5° between the ages of 20–60 years, which is due to increasing HVA or deformation of the metatarsal head caused by repeated stress. In 2018, Frumberg et al. [7] performed a first metatarsal (M1) osteotomy in a cadaver study and compared the DMAA measured by radiographs before and after the correction of metatarsal rotation, finding that M1 rotation affects the accuracy of DMAA measurement.

Dayton et al. [5] recognizes that M1 pronation and subluxation of the sesamoid are important components of HV deformity and must be repaired during the surgery to provide basic anatomical alignment. With weight-bearing anteroposterior radiographs of the foot, Hardy et al. [8] classifies the position of the medial sesamoid into seven grades based on the relationship of the medial sesamoid to the central axis of M1. In recent years, it has been revealed that tangential sesamoid radiographs, which show the actual status of the sesamoid to the metatarsal head more clearly, can be used to assess not only whether the position of the sesamoid relative to the metatarsal head is matched or displaced, but also the axial rotation of the M1 head. Yildirim et al. [21] has established a four-stage grading system according to the position of the medial sesamoid relative to the intersesamoid ridge. In a weight-bearing CT (WBCT) study, Kim et al. [10] found that the α angle, which reflects the rotation of M1, was 13.8° in the control group compared with 21.9° in the HV group, and the angle values were positively correlated with the sesamoid position. However, Shibuya et al. [17] found that the first metatarsal rotation angle (MRA) was not correlated to the sesamoid position after adjustment for the covariates.

According to these concepts, we hypothesized that the increase in DMAA and the subluxation of the sesamoid were related to the rotation of M1, which might be secondary to soft tissue fatigue. Therefore, we conducted a retrospective self-controlled study with unilateral HV patients to validate our hypothesis.

## Methods

**Fig. 1:**
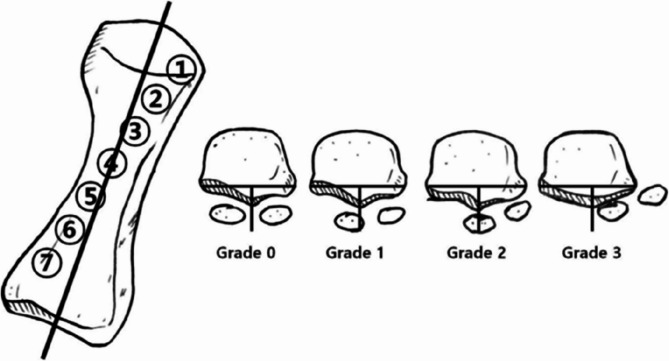
Hardy score in weight-bearing anteroposterior radiograph and Yildirim score in tangential sesamoid radiograph

This study was a single-center, retrospective, self-controlled design and conducted at Huashan Hospital affiliated to Fudan University. The use of the contralateral healthy foot as an internal control helped minimize confounding biases caused by individual anatomical variations, improving intergroup comparability. Bilateral weight-bearing anteroposterior radiographs of the foot and tangential sesamoid radiographs were obtained in patients with unilateral HV admitted to our hospital between January 2015 and December 2018. The affected and contralateral normal feet were divided into HV and normal groups. The following parameters were measured and compared between the two groups: HVA, DMAA, IMA, MRA, Hardy score, and Yildirim score in two groups (Fig. 1).

### Inclusion and exclusion criteria

Patients were included according to the following criteria: (1) adults over 18 years old; (2) congenital unilateral hallux valgus; (3) no other lower limb-related disorders. Patients were excluded according to the following criteria: (1) bilateral hallux valgus; (2) traumatic or secondary hallux valgus; (3) other lower limb-related disorders, such as flatfoot, ankle deformity and knee/ankle arthritis.

### Parameter measurements

**Fig. 2:**
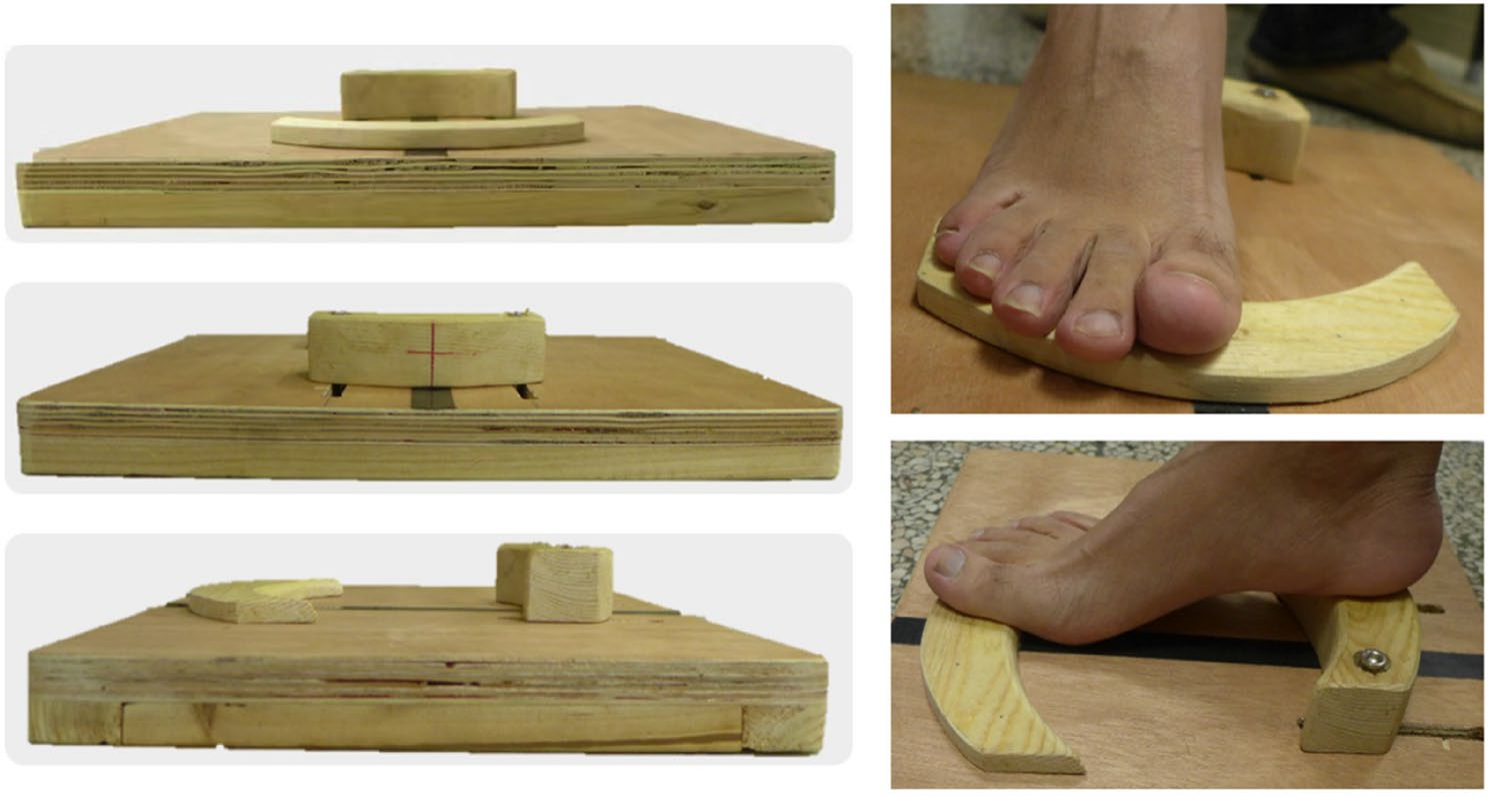
The wooden platform and foot position for tangential sesamoid radiographs

**Fig. 3:**
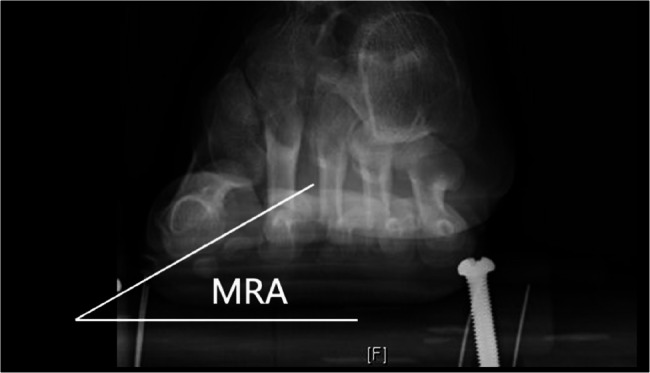
MRA in tangential sesamoid radiograph

All radiographic measurements were independently performed by two experienced orthopedic surgeons. The average of two measurements was taken as the final result. The value of HVA, IMA, DMAA, and Hardy score was obtained in weight-bearing anteroposterior radiographs, and the value of Yildirim score was measured in tangential sesamoid radiograph. Tangential sesamoid radiographs were obtained by placing the patient’s heel and forefoot on differential wooden platforms of varying heights (Fig. 2). The MRA was the angle between the horizontal line and a line connecting the deepest point of the sesamoid groove on both sides in tangential sesamoid radiograph (Fig. 3) to reflect the rotation of M1 [17].

### Statistical analysis

The HVA, IMA, DMAA and MRA values of the two groups were statistically compared using a paired *t*-test, and the Hardy score and Yildirim score were compared using Cochrane Q test. The correlation between the MRA and DMMA of the HV group was assessed using Pearson correlation coefficient, and the correlation between MRA and Hardy score and Yildirim score of the HV group was assessed with Spearman correlation coefficient. Inter- and intraobserver reliabilities of the parameters were assessed with the intraclass correlation coefficient (ICC). Statistical analysis was performed with SPSS 17.0 (IBM, Chicago, IL, USA). P values < 0.05 were considered statistically significant.

## Results

**Fig. 4:**
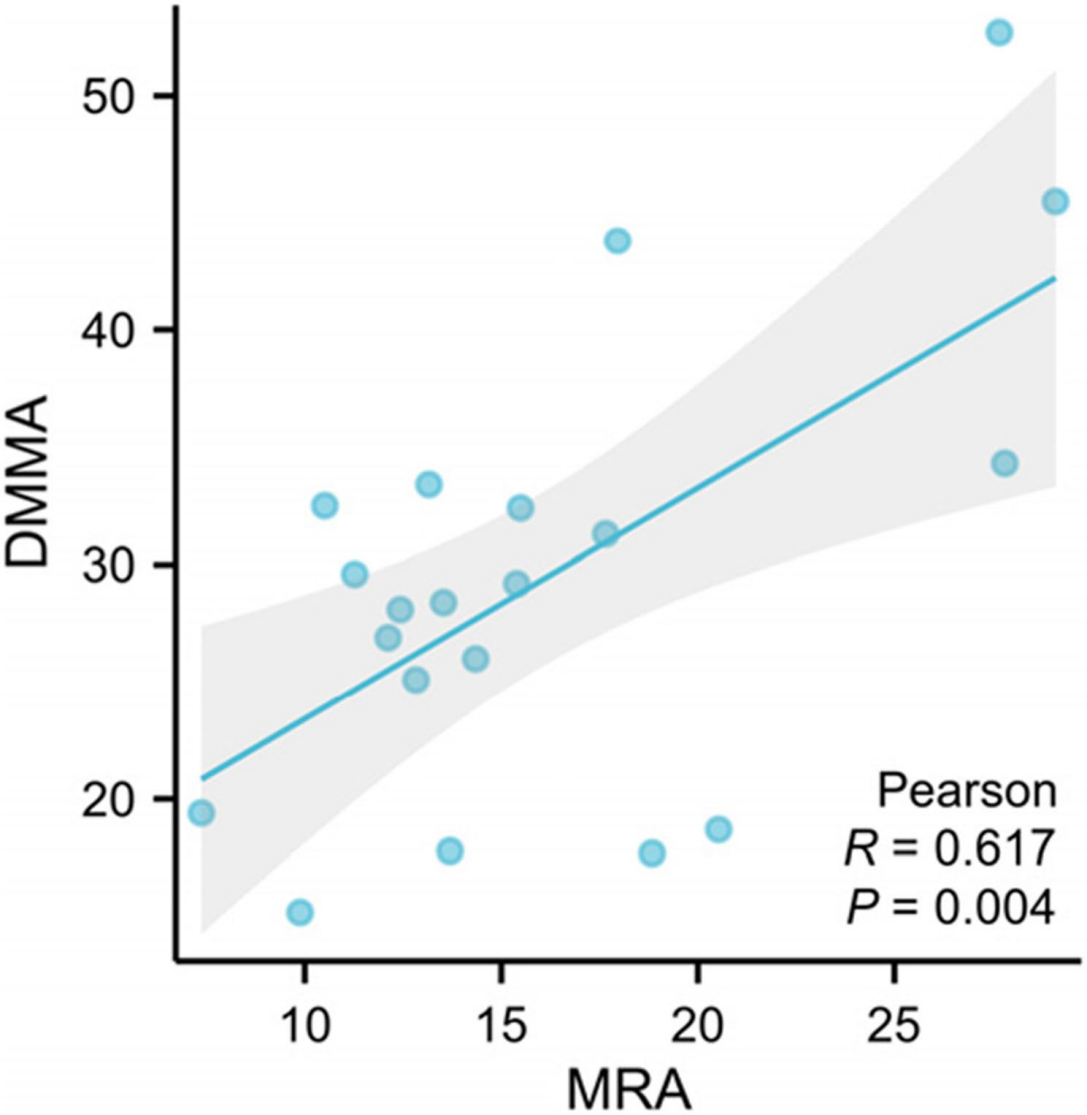
Correlation analysis between MRA and DMAA

**Fig. 5:**
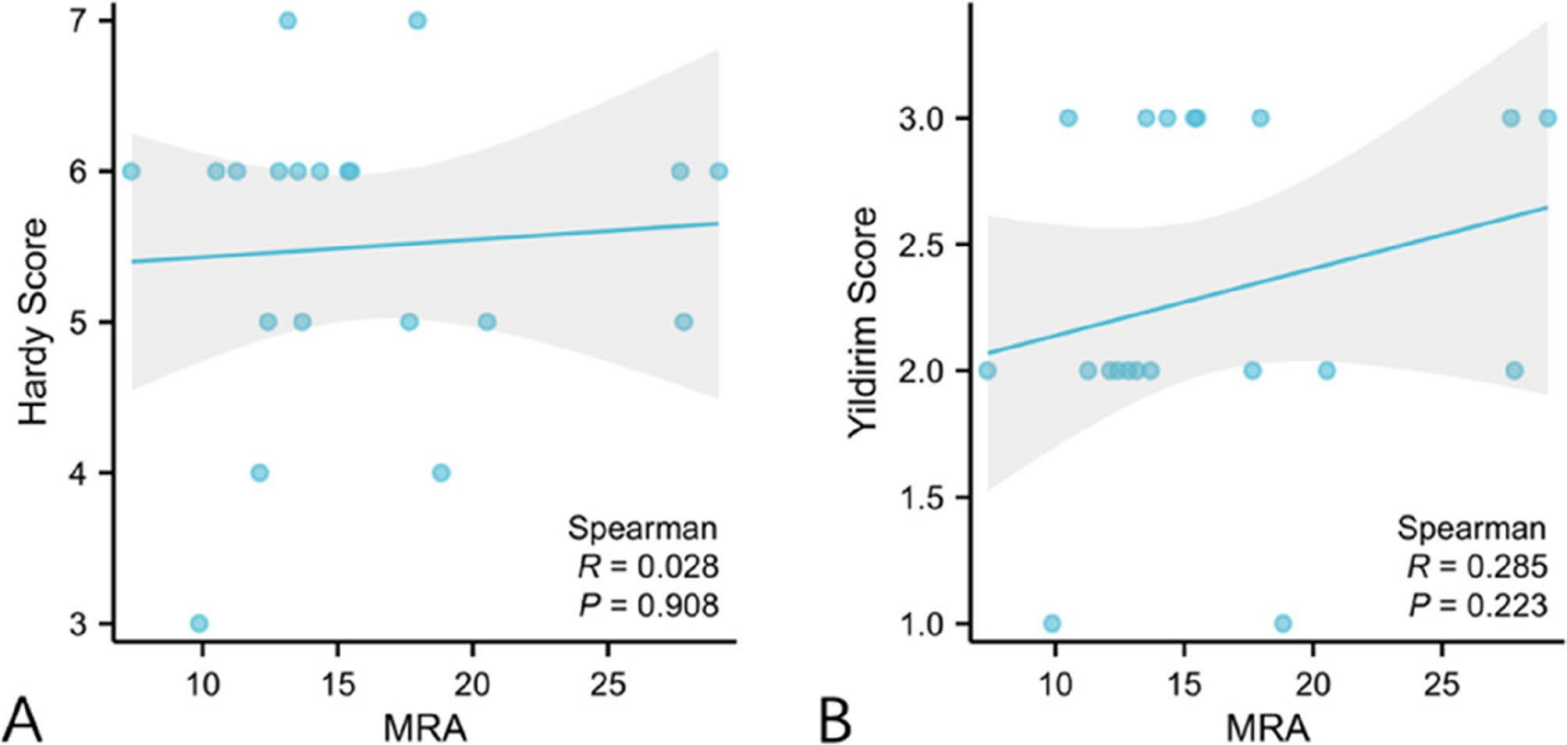
Correlation analysis between MRA, Hardy and Yildirim score. **A** MRA and Hardy score, (**B**) MRA and Yildirim score

From January 2015 to December 2018, there were 20 unilateral HV patients, who satisfied the inclusion criteria, undergoing surgery in our hospital in total. These 20 patients, comprising 1 male and 19 females, were enrolled in the study, with a mean age of 53.35 ± 13.90 years (range 25–73). There were 20 feet in the HV group, with a mean HVA of 40.04 ± 8.96°, a mean DMAA of 29.40 ± 9.73°, a mean IMA of 13.34 ± 2.73°, a mean MRA of 16.08 ± 6.10, a mean Hardy score of 5.5 ± 0.97, and a mean Yildirim score of 2.35 ± 0.65 (Table 1). There were also 20 feet in the normal group, with a mean HVA of 16.06 ± 2.70°, a mean DMAA of 16.13 ± 7.77°, a mean IMA of 10.02 ± 2.14°, a mean MRA of 9.56 ± 3.81, a mean Hardy score of 3.4 ± 1.02, and a mean Yildirim score of 0.85 ± 0.73. The HVA, IMA, DMAA, MRA, Hardy and Yildirim score were significantly higher in the HV group than in the normal group (*P* < 0.001) (Table 1). Intra- and interobserver reliabilities of each parameter measurements showed excellent consistency (Table 2). The MRA was positively correlated with the DMAA (*r* = 0.617, *P* = 0.004), but was not correlated with Hardy score (*r* = 0.028, *P* = 0.908), and Yildirim score (*r* = 0.285, *P* = 0.223) (Figs. 4 and 5).

**Table 1:** Hallux valgus group and normal group comparison

	HV Group (*n* = 20) (mean ± SD)	Normal Group (*n* = 20) (mean ± SD)	*P* Value
HVA, degree	40.04 ± 8.96	16.06 ± 2.70	< 0.001
IMA, degree	13.34 ± 2.73	10.02 ± 2.14	< 0.001
DMAA, degree	29.40 ± 9.73	16.13 ± 7.77	< 0.001
MRA, degree	16.08 ± 6.10	9.56 ± 3.81	< 0.001
Hardy score	5.5 ± 0.97	3.4 ± 1.02	< 0.001
Yildirim score	2.35 ± 0.65	0.85 ± 0.73	< 0.001

*Abbreviations*: *HV* hallux valgus, *HVA* hallux valgus angle, *IMA* intermetatarsal angle, *DMAA* distal metatarsal articular angle,* MRA* first metatarsal rotation angle

**Table 2:** Intra- and interobserver reliabilities of each parameter measurements

	Intraobserver Reliability ICC (95% CI)	Interobserver Reliability ICC (95% CI)
HVA	0.98 (0.96–0.99)	0.96 (0.92–0.98)
IMA	0.96 (0.92–0.98)	0.95 (0.90–0.98)
DMAA	0.92 (0.84–0.96)	0.93 (0.86–0.97)
MRA	0.91 (0.82–0.96)	0.89 (0.78–0.95)
Hardy score	0.95 (0.90–0.98)	0.94 (0.88–0.97)
Yildirim score	0.92 (0.84–0.96)	0.90 (0.80–0.95)

*Abbreviations*: *ICC* intraclass correlation coefficient, *CI* confidence interval, *HV* hallux valgus, *HVA* hallux valgus angle, *IMA* intermetatarsal angle, *DMAA* distal metatarsal articular angle, *MRA* first metatarsal rotation angle

## Discussion

This study specifically included patients with unilateral hallux valgus to utilize their contralateral healthy feet as an internal control. This design effectively controlled the inter-individual anatomical and physiological variations, providing a unique advantage for analyzing the relationship between MRA and DMAA. Although unilateral deformity is not epidemiologically predominant and is often associated with secondary factors, this study implemented strict exclusion criteria to get an ideal homogeneous cohort for further analysis. The key findings demonstrated significant differences in all measured parameters—HVA, IMA, DMAA, MRA, Hardy score, and Yildirim score—between the HV and normal groups. The principal findings of this study corroborated our initial hypothesis by demonstrating a significant positive correlation between the M1 rotation, quantified by the MRA, and the DMAA in patients with HV. However, our study did not reveal a significant correlation between MRA and the sesamoid position as graded by the Hardy and Yildirim classification systems.

 The significant elevation of HVA, IMA, DMAA, MRA, and both sesamoid scores in the HV group aligned with the understanding of HV as a complex three-dimensional deformity [6]. Several studies have found that after the correction of M1 rotation, DMAA can be significantly decreased [4, 7, 15]. Therefore, it has been argued that DMAA can only reflect the rotation of the metatarsal and is not related to the deformity of the distal articular surface [4, 18]. In the study of Lalevée et al., the mean difference in DMAA on radiographs was 18.4 degrees, which was 13.27 degrees in our study, while the difference was reduced to 8.6 degrees after computerized correction of pronation [11]. It suggested that DMAA was not only a radiographic consequence of M1 rotation, but also correlated with metatarsal head deformation [11]. Our results were consistent with the cadaver study of Robinson et al. [15], who demonstrated that M1 pronation could directly influence the measurement of DMAA. The moderate positive correlation (*r* = 0.617, *p* = 0.004) we observed provided clinical evidence supporting the biomechanical link between these two parameters in vivo. This suggested that rotational deformity of the first metatarsal was not an isolated phenomenon but was integrally related to the degeneration of the articular surface of the M1 [11]. Wang et al. also found that the incongruency of the first metatarsophalangeal joint was related to larger DMAA [20]. These findings have direct implications for surgical planning. It is crucial to conduct a thorough preoperative evaluation of M1 rotation. Furthermore, the assessment of correcting the M1 pronation may be imperative if an inadequate correction of the DMAA is observed during surgery.

In contrast to our hypothesis, our study revealed an absence of significant correlation between MRA and the established sesamoid grading systems. While both the Hardy and Yildirim scores were significantly worse in the HV group, their values did not linearly correlate with the degree of M1 rotation. This finding was in line with the outcome of some researches. Lalevée et al. found that there was no significant difference in M1 pronation between sesamoid grade 2 and grade 3 [12]. In a WBCT study of Kim et al., they identified cases with M1 pronation without the presence of sesamoid displacement, which was named pseudo-sesamoid subluxation [10]. This discrepancy could be interpreted from several perspectives. First, it supported the perspective proposed by Shibuya et al. [17] that the relationship between rotation and sesamoid position may be more complex than a simple linear correlation and could be influenced by other covariates, such as the integrity of the medial soft-tissue restraints or the duration of the deformity [12]. Second, and more critically, it may highlight a fundamental limitation of conventional two-dimensional radiographic grading systems. They may lack the sensitivity to specifically quantify the rotational component of the deformity. The sesamoid grading system does not take the metatarsal pronation into consideration, which is an important factor to evaluate the true condition of sesamoid subluxation [10]. Our findings resonated with the evolving understanding that advanced three-dimensional imaging, such as WBCT, was superior for precisely delineating the relationship between bony rotation and sesamoid articulation [11]. The tangential sesamoid radiograph, while providing a clearer view than the AP projection, may still project a three-dimensional relationship onto a two-dimensional plane, potentially obscuring the influence of M1 rotation 

 There are several limitations in our study. Firstly, in previous study, it was found that the DMAA in HV patients with joint incongruency was larger and the M1 rotation was also related to the join congruency [20]. However, due to the relatively small sample size, we did not investigate the influence of the first metatarsophalangeal joint congruency in this study. Further study is needed to take it into consideration. Secondly, we did not include juvenile HV patients, whose changes in developmental DMAA were not investigated in our study. Several studies have found that the early onset of juvenile HV is characterized by an increased DMAA, and the success of the surgery is highly correlated with the magnitude of the DMAA [1, 9]. It is necessary to include juvenile patients for further study. Finally, the implementation of WBCT for the study was not feasible due to prevailing conditions. The values of DMAA on conventional radiographs may be overestimated [11] and the interobserver agreement of DMAA measurement on CT 3D reconstruction can be increased, which may have an impact on the results [3, 13].

## Conclusion

 This study confirmed that first metatarsal rotation was a significant component of HV deformity and was positively correlated with DMAA. This reinforces the notion that a comprehensive preoperative evaluation should include an assessment of rotational alignment. However, the absence of correlation with traditional sesamoid scores suggested that these grading systems may be inadequate for fully characterizing the rotational pathology. Ultimately, it is essential to thoroughly evaluate the rotation deformity preoperatively and determine the necessity of concomitant M1 derotation when the restoration of DMAA is inadequate.

## Data Availability

The datasets used and analyzed during the current study are available from the corresponding author on reasonable request.

## Abbreviations

DMAA: Distal metatarsal articular angle
HV: Hallux valgus
HVA: Hallux valgus angle
IMA: Intermetatarsal angle
M1: First metatarsal
MRA: First metatarsal rotation angle
WBCT: Weight-bearing CT
